# Clinical efficacy and safety of astragalus injection combined with ACEI/ARB in the treatment of diabetic kidney disease: Protocol for a systematic review and meta-analysis

**DOI:** 10.1097/MD.0000000000031490

**Published:** 2022-12-09

**Authors:** Zhiyue Zhu, Qi Zhang, Le Liu, Pengjie Bao, Shilin Liu, Chaoqun Song, Wenbo Yang, Zheng Nan

**Affiliations:** a College of Chinese Medicine, Changchun University of Chinese Medicine, Changchun, China; b Institution of Shenzhen Hospital, Guangzhou University of Chinese Medicine (Futian), Shenzhen, China; c Department of Pediatrics, Affiliated Hospital of Jiangxi University of Chinese Medicine, Nanchang, China.

**Keywords:** ACEI/ARB, astragalus injection, diabetic kidney disease, meta analysis, protocol

## Abstract

Kidney disease is a common complication of diabetes and the main cause of end-stage renal disease. Astragalus (Huangqi) injection in combination with angiotensin-converting enzyme inhibitors/angiotensin receptor blockers (ACEI/ARBs) have been widely used for the treatment of diabetic kidney disease (DKD) in China. However, no supporting evidence yet exists with regard to the safety and effectiveness of this approach. Here a protocol is outlined for use in systematic evaluation of the safety and effectiveness of astragalus injection combined with ACEI/ARB for the treatment of DKD.

**Methods:**

Randomised controlled trials will be retrieved from 8 scientific databases, including PubMed, Web of Science, EMBASE database, Cochrane Library, China National Knowledge Infrastructure, Wanfang, China Biomedical Literature CD-ROM Database and China Science Journal Database. Ongoing clinical trial databases will also be searched for studies published from the time of establishment of each database to September 1, 2022. that will include the Chinese Clinical Trial Registration Centre (www.chictr.org.cn/), the World Health Organisation International Trial Registration Platform (https://www.who.int/clinical-trials-registry-platform), Google Scholar (https://scholar.google.com/), Baidu Scholar (https://xueshu.baidu.com), etc. The main outcome indicators included urinary albumin excretion rate or 24-hour urinary albumin excretion rate, and renal function (blood urea nitrogen, serum creatinine concentration). The secondary outcome indicators mainly include the following 4 aspects: blood sugar, blood pressure, blood lipid levels and adverse events. Two researchers will independently select and extract data from randomized controlled trials and determine risks of bias. Meta-analysis will be performed using Revman5.4 then the quality of evidence from randomized clinical trials will be assessed using the Grading of Recommendations Assessment, Development and Evaluation (GRADE) System tool.

**Results:**

This review will be the first to summarize meta-analysis results regarding the efficacy and safety of Huangqi injection combined with ACEI/ARB when administered during any stage of diabetic nephropathy rather than during only a single stage of the disease.

**Discussion:**

It will provide high-quality guidance for the treatment of diabetic kidney disease and provide patients with more treatment options.

## 1. Introduction

Diabetic kidney disease (DKD), a microvascular disorder caused by renal glomerular and tubular dysfunction, is a major diabetic complication^[1]^ and a major cause of end-stage renal disease (ESRD).^[2]^ DKD prevalence is increasing in step with the continual increase in number of diabetic patients worldwide and is projected to afflict an estimated 700 million people by 2045.^[3]^ As compared with traditional diabetic nephropathy, DKD includes a wider range of renal disorders that includes impaired renal function without proteinuria.^[4]^ The International Diabetes Federation (IDF) has reported that about 40% of diabetic patients will develop ESRD.^[5]^ At the same time, DKD often leads to development of cardiovascular disease.^[6]^ Thus, DKD is a serious global challenge that will impose ever increasing long-term treatment costs upon individuals and society until a cure is found for the disease.^[7]^

Development of DKD is related to many factors, including heritable factors that have been revealed through studies of family disease clusters^[8]^ resulting in identification of several specific DKD-associated genes.^[9]^ In addition, metabolic factors, haemodynamic factors, growth factors, and pro-inflammatory or pro-fibrotic factors all play key roles in DKD incidence,^[10]^ with chronic inflammation considered a major feature of DKD.^[11]^ Nevertheless, while identifying causes of DKD is important, treatments to prevent or alleviate the disease are urgently needed since no cure yet exists for the disease.^[3]^ Towards this end, studies have shown that strengthening of early blood sugar control can play an important role in preventing DKD.^[12]^ Moreover, several other strategies are currently used to slow DKD development, such as lowering blood sugar, adjusting dietary protein intake,^[13]^ treating hypertension with renin angiotensin-aldosterone system (RAAS) blockers,^[14]^ etc. Furthermore, angiotensin-converting enzyme inhibitor (ACEI) or angiotensin II receptor antagonist (ARB), which can reduce urinary albumin levels to protect the kidneys and slow DKD development,^[15]^ are commonly used as clinical treatments for DKD. However, administration of ACEI/ARB cannot prevent DKD development, prompting researchers to search for more effective treatments to prevent the disease.

Although many new drugs and treatment approaches are in current use for the clinical treatment of DKD, their clinical efficacies are still unclear. Astragalus (Huangqi) injection, which is widely used in China, is a traditional Chinese medicine that plays an important role in DKD treatment by fighting inflammation and preventing kidney damage.^[16]^ Intriguingly, pharmacological studies conducted in with rats streptozotocin (STZ)-induced diabetic disease have shown that astragalus can reduce proteinuria and urea nitrogen levels,^[17]^ while also exerting unique curative effects during different DKD stages.^[18]^ However, most previously conducted meta-analyses have only investigated the efficacy of astragalus injection administered during early DKD, even though this treatment has been shown to improve immunity of dialysis patients in renal failure.^[19]^ Furthermore, in China astragalus injection is not frequently used alone to treat DKD, but is usually combined with ACEI/ARB to achieve good clinical effect. Meanwhile, although several systematic reviews have evaluated efficacies in DKD patients in a single, specific disease stage or have evaluated astragalus injection alone, comprehensive evidence-based evaluations of Huangqi injection combined with ACEI/ARB for the treatment of DKD are still urgently needed to guide the development of optimal treatment plans for use in patients in different DKD stages.

## 2. Methods

### 2.1. Design and registration of the review

This systematic review and meta-analysis protocol has been registered with PROSPERO (registration number: CRD42022300652). The protocol was developed based on System Review and Meta-Analysis Programme Preferred Reporting Items, as outlined in the Systematic Review and Meta-Analysis Protocols (PRISMA-P) Preferred Reporting Project guidelines.

### 2.2. Inclusion criteria

Eligibility criteria will be based on the population, intervention, comparison, outcomes and study (PICOS) design framework. Research will adhere to criteria with regard to research type, participants, interventions, controls and types of measured outcomes (results) as outlined in Table 1.

**Table 1:** PICOS data elements.

Type of studies	ΔRandomized controlled trials (RCT)
	ΔThe search deadline: September 1, 2022
Population	ΔSample size
	ΔAge
	ΔGender
	ΔWithdrawals
Intervention/Comparator	ΔIntervention
	ΔTiming
	ΔFrequency of intervention
	ΔNo of sessions
Outcomes	ΔThe primary outcomes indicators: urinary albumin excretion rate, 24 h urinary albumin excretion rate
	ΔThe secondary outcome indicators: renal function (blood urea nitrogen, serum creatinine concentration), blood sugar, blood pressure, blood lipid levels and adverse events
	ΔAdverse events related to the intervention, including but not limited to: headache, weakness, nausea, vomiting, abnormal liver function, hypo/hypertension

#### 2.2.1. Types of studies.

We will include randomized controlled trials of Huangqi injection combined with ACEI/ARB for the treatment of DKD in this systematic review and meta-analysis. All other types of research will be excluded, such as animal studies, cohort studies, cross-sectional studies, case-control studies, etc, as well as repeated studies. Studies of Astragalus injection that are not for injectable use (e.g., oral use) will also be excluded.

#### 2.2.2. Characteristics of participants.

The group of study participants will be comprised of adults diagnosed with DKD as based on clear clinical diagnostic criteria (patients with diabetes based on the presence of albuminuria and/or reduced eGFR in the absence of signs or symptoms of other primary causes of kidney damage. The typical presentation of DKD is considered to include a long-standing duration of diabetes, retinopathy, albuminuria without gross hematuria, and gradually progressive loss of eGFR, or articles that clearly state that DKD is diagnosed according to guidelines, such as the Chinese Guidelines for the Clinical Treatment of DKD), including patients in all stages of DKD regardless of age, gender, nationality and race.

#### 2.2.3. Types of interventions and controls.

The control group will include patients treated with ACEI/ARB. The intervention group will include patients treated with Huangqi injection in combination with ACEI/ARB.

#### 2.2.4. Types of measured outcomes.

Primary outcomes indicators will include urinary albumin excretion rate or 24-hour urinary albumin excretion rate. Secondary outcomes indicators will mainly include blood urea nitrogen, serum creatinine concentration, fasting blood glucose, 2-hour postprandial blood glucose, glycosylated hemoglobin, blood pressure, triglyceride, total cholesterol and adverse events.

### 2.3. Exclusion criteria

Exclusion criteria will include duplicate publications or identical data reported in different papers in the literature, clinical studies without clear diagnostic criteria, studies of Astragalus injection that are not for injectable use (e.g., oral use),incomplete original data, studies of kidney damage caused by diseases other than diabetes, specialized patient groups (e.g., lactating women, pregnant women), patients with other serious systemic diseases and clinical studies lacking rigorous scientific design (lack of a control group, incorrectly applied statistical methods, etc).

### 2.4. Search strategy

We will comprehensively search 4 English medical databases and 4 Chinese databases: PubMed, Web of Science, EMBASE database, Cochrane Library, China National Knowledge Infrastructure, Wanfang, China Biomedical Literature CD-ROM Database, China Science Journal Database. The search time frame will be defined as the time period between the establishment of the database to the end date of September 1, 2022. Meanwhile, in order to obtain comprehensive access to relevant clinical research trials, ongoing clinical trial databases will also be searched, such as the Chinese Clinical Trial Registration Centre (www.chictr.org.cn/), the World Health Organisation International Trial Registration Platform (https://www.who.int/clinical-trials-registry-platform),Google Scholar (https://scholar.google.com/), Baidu Scholar (https://xueshu.baidu.com), etc. The search strategy used for PubMed is shown in Table 2.

**Table 2:** Search strategy for the PubMed database.

Number	Terms
#1	Diabetic Nephropathies[Mesh]
#2	((((((((((((((((Nephropathies, Diabetic[Title/Abstract]) OR (Nephropathy, Diabetic[Title/Abstract])) OR (Diabetic Nephropathy[Title/Abstract])) OR (Diabetic Kidney Disease[Title/Abstract])) OR (Diabetic Kidney Diseases[Title/Abstract])) OR (Kidney Disease, Diabetic[Title/Abstract])) OR (Kidney Diseases, Diabetic[Title/Abstract])) OR (Diabetic Glomerulosclerosis[Title/Abstract])) OR (Glomerulosclerosis, Diabetic[Title/Abstract])) OR (Intracapillary Glomerulosclerosis[Title/Abstract])) OR (Nodular Glomerulosclerosis[Title/Abstract])) OR (Glomerulosclerosis, Nodular[Title/Abstract])) OR (Kimmelstiel–Wilson Syndrome[Title/Abstract])) OR (Kimmelstiel–Wilson Syndrome[Title/Abstract])) OR (Syndrome, Kimmelstiel–Wilson[Title/Abstract])) OR (Kimmelstiel–Wilson Disease[Title/Abstract])) OR (Kimmelstiel–Wilson Disease[Title/Abstract])
#3	#1 OR #2
#4	Astragalus Injection[Title/Abstract]
#5	Astragalus[Title/Abstract]
#6	#4 OR #5
#7	Angiotensin Receptor Antagonists[Mesh]
#8	(((((((((((((Antagonists, Angiotensin Receptor[Title/Abstract]) OR (Receptor Antagonists, Angiotensin[Title/Abstract])) OR (Angiotensin Receptor Blockers[Title/Abstract])) OR (Receptor Blockers, Angiotensin[Title/Abstract])) OR (Angiotensin Receptor Blocker[Title/Abstract])) OR (Blocker, Angiotensin Receptor[Title/Abstract])) OR (Receptor Blocker, Angiotensin[Title/Abstract])) OR (Angiotensin Receptor Antagonist[Title/Abstract])) OR (Antagonist, Angiotensin Receptor[Title/Abstract])) OR (Receptor Antagonist, Angiotensin[Title/Abstract])) OR (Angiotensin II Receptor Antagonists[Title/Abstract])) OR (Angiotensin II Receptor Blockers[Title/Abstract])) OR (Angiotensin II Receptor Antagonist[Title/Abstract])) OR (Angiotensin II Receptor Blocker[Title/Abstract])
#9	#7 OR #8
#10	Angiotensin-Converting Enzyme Inhibitors[Mesh]
#11	(((((((((((((((((((((((((((Angiotensin Converting Enzyme Inhibitors[Title/Abstract]) OR (Enzyme Inhibitors, Angiotensin-Converting[Title/Abstract])) OR (Inhibitors, Angiotensin-Converting Enzyme[Title/Abstract])) OR (Inhibitors, Angiotensin Converting Enzyme[Title/Abstract])) OR (Inhibitors, Kininase II[Title/Abstract])) OR (Kininase II Antagonists[Title/Abstract])) OR (Kininase II Inhibitors[Title/Abstract])) OR (Angiotensin-Converting Enzyme Antagonists[Title/Abstract])) OR (Angiotensin Converting Enzyme Antagonists[Title/Abstract])) OR (Enzyme Antagonists, Angiotensin-Converting[Title/Abstract])) OR (Kininase II Inhibitor[Title/Abstract])) OR (II Inhibitor, Kininase[Title/Abstract])) OR (Inhibitor, Kininase II[Title/Abstract])) OR (Antagonists, Kininase II[Title/Abstract])) OR (Inhibitors, ACE[Title/Abstract])) OR (ACE Inhibitors[Title/Abstract])) OR (Angiotensin I-Converting Enzyme Inhibitors[Title/Abstract])) OR (Angiotensin I Converting Enzyme Inhibitors[Title/Abstract])) OR (Angiotensin Converting Enzyme Inhibitor[Title/Abstract])) OR (ACE Inhibitor[Title/Abstract])) OR (Inhibitor, ACE[Title/Abstract])) OR (Angiotensin I-Converting Enzyme Inhibitor[Title/Abstract])) OR (Angiotensin I Converting Enzyme Inhibitor[Title/Abstract])) OR (Angiotensin-Converting Enzyme Inhibitor[Title/Abstract])) OR (Enzyme Inhibitor, Angiotensin-Converting[Title/Abstract])) OR (Inhibitor, Angiotensin-Converting Enzyme[Title/Abstract])) OR (Antagonists, Angiotensin-Converting Enzyme[Title/Abstract])) OR (Antagonists, Angiotensin Converting Enzyme[Title/Abstract])
#12	#10 OR #11
#13	#9 OR #12
#14	Randomized controlled trial[Publication Type] OR randomized[Title/Abstract] OR placebo[Title/Abstract]
#15	#3 AND #6 AND #13 AND #14

### 2.5. Research study selection

**Figure 1.**
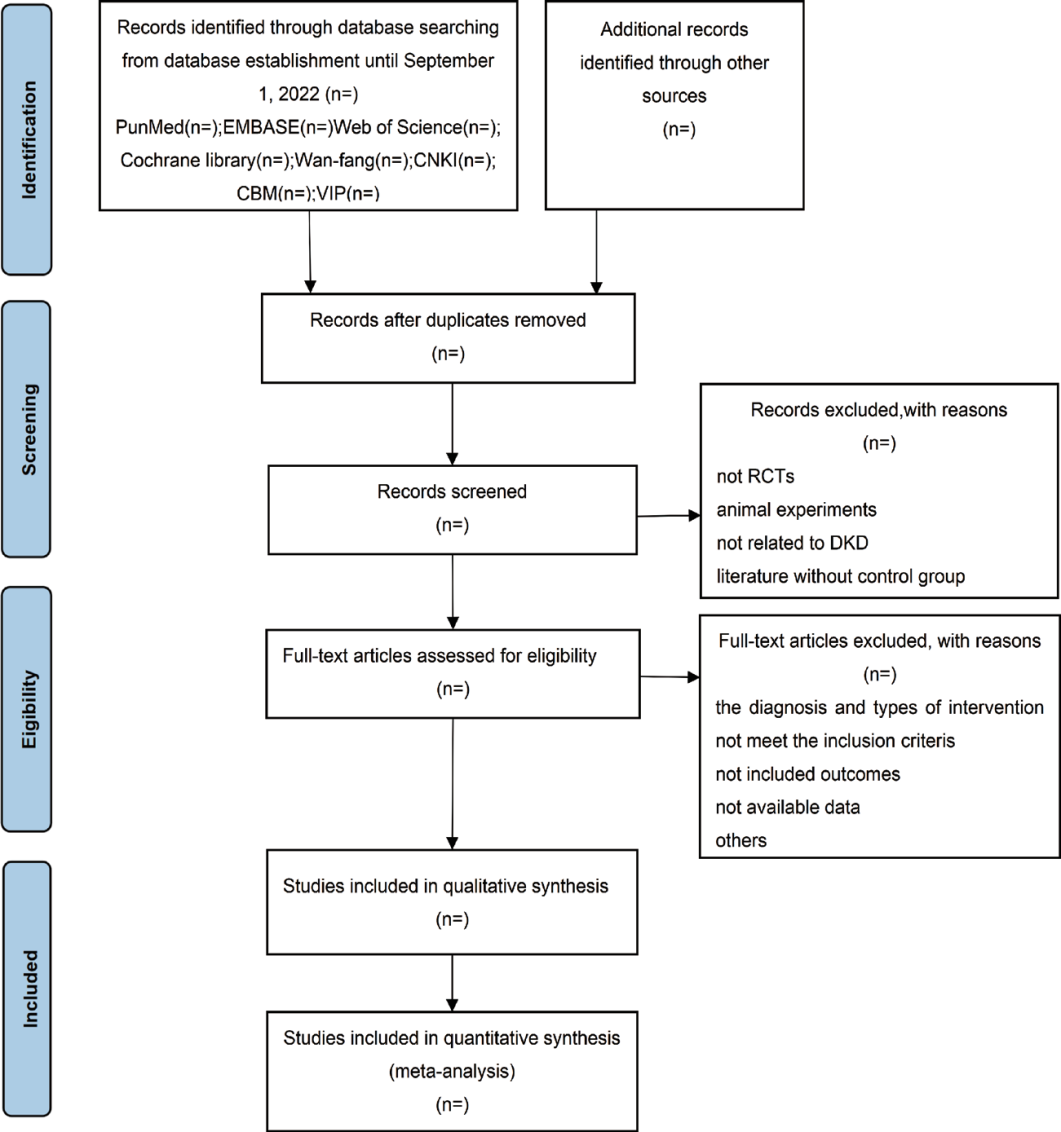
Flow diagram of study selection process.

We will import the final retrieved data into Endnote X9 for document management. After excluding duplicate study reports, 2 independent reviewers will screen the remaining studies based on previously designed inclusion and exclusion criteria in order to exclude non-conforming studies. Reasons for exclusion of studies will be documented. In the event that disagreement between reviewers occurs, the issue will be discussed to resolve the disagreement or will be resolved by consensus based on screening results obtained by a third reviewer. This literature screening protocol meets PRISMA standards and is summarized in Figure 1.

### 2.6. Data extraction

Two independent reviewers will enter the final set of selected data into the EXCEL form, including the first author of the study, publication year, participant characteristics, intervention measures, outcome indicators and adverse events (Table 3). Thereafter, 2 independent reviewers will cross-check entered data to ensure data accuracy and validity. If there is a lack of original data for a study at that time, we will contact the original author via email. Thereafter, if the required information cannot be obtained, the study will be excluded.

**Table 3:** General information of recruited studies.

Reference	Sample	Design	Age	Gender	Intervention	Comparator	UAER	BUN	Scr	FBG	2hPBG	HbA1c	BP	TG	TC	Adverse events
Authors, yr	Number of participants	RCT	Mean (SD), yr	Male/female	Astragalus injection combined with ACEI/ARB	ACEI/ARB	Mean (SD), μg/min	Mean (SD), mmol/L	Mean (SD), μmol/L	Mean (SD), mmol/L	Mean (SD), mmol/L	Mean (SD), %	Mean (SD), mm Hg	Mean (SD), mmol/L	Mean (SD), mmol/L	Headache,weakness, nausea,vomiting, abnormal liver function,et

2hPBG = 2-hr postprandial blood glucose, ACEI = angiotensin-converting enzyme inhibitor, ARB = angiotensin II receptor antagonist, BP = blood pressure, BUN = blood urea nitrogen, FBG = fasting blood glucose, HbA1c = glycosylated hemoglobin, Scr = serum creatinine concentration, TC = total cholesterol, TG = triglyceride, UAER = urinary albumin excretion rate or 24-hr urinary albumin excretion rate.

### 2.7. Bias evaluation of included studies

Due to the fact that only randomized controlled trials will be included, selected studies will be evaluated using the Cochrane risk of bias assessment tool, with separate evaluations conducted based on the following 6 criteria: random allocation method, allocation plan concealment, blind method, result data completeness, selective reporting of research results, and other sources of bias. Finally, the evaluation results will be summarized and assigned to categories including high risk of bias, uncertain risk of bias and low risk of bias. Results will be evaluated by 2 researchers. If disagreements occur, they will be discussed or resolved by consensus based on input from a third reviewer. This information will also be provided in the research report with reasons underlying the final judgement provided in the “risk of bias” table.

### 2.8. Data integration and analysis

We will use Revman 5.4 to conduct meta-analysis. Binary variables will be calculated and expressed as the risk ratio and 95% CI, while continuous variables will be calculated and expressed as the mean difference and 95% CI. For evaluation of heterogeneity across studies, results will mainly be evaluated using the χ^2^ test and the inconsistency index (*I*^2^). If heterogeneity is detected based on *I*^2^ ≥ 50% and *P* < .1, then factors associated with study heterogeneity will be identified (e.g., differences in research subject characteristics, drug dosages across studies, etc) using subgroup analysis and sensitivity analysis. Subgroup analysis will be used as needed to group different influences, such as different stages of DKD, according to the observed heterogeneity, while sensitivity analysis will be used to help screen out studies with high risks of bias. Ultimately, if *I*^2^ ≥ 50% and *P* < .1 are both found to be true (thus indicating marked heterogeneity) and underlying reasons for heterogeneity cannot be identified, the random effects model will be used for data analysis. Otherwise, the fixed effects model will be used.

### 2.9. Assessment of publication bias

After screening of studies is complete for a final number of included studies that is >10, we will judge whether publication bias exists based on whether the funnel plot is symmetrical. In addition, the heterogeneous treatment of data involved in meta-analysis and other potential sources of bias will be described.

### 2.10. Grading of the quality of evidence collected

The quality of the research evidence will be evaluated using the recommended Grading Evaluation, Development and Evaluation (GRADE) method.^[20]^ We will base our selection of evidence on whether limitations exist in the design and implementation of the research (methodological quality), whether the evidence is indirect, whether unexplainable heterogeneity exists in the results, whether the results are accurate, whether publication bias exists, etc. Thereafter, the evidence will be assigned to categories including high-level evidence, intermediate-level evidence, low-level evidence or very low-level evidence.

### 2.11. Ethics and communication

Studies included in our analysis will have been published publicly and thus will not require ethical approval for inclusion in the study. We will publish the results of this research in a peer-reviewed journal.

### 2.12. Patient and public involvement

There will be no patient or public participation in this study.

## 3. Discussion

DKD, the leading cause of diabetic patient mortality worldwide, is increasing in prevalence due to increasing numbers of diabetic patients, with increases most marked in developing countries. Meanwhile, in developed countries DKD is currently the single greatest cause of ESRD.^[21]^ Due to the complex pathogenesis of DKD, conventional blood pressure and blood sugar control therapies cannot prevent DKD progression to ESRD,^[22]^ thus highlighting the urgent need for new treatments to prevent DKD progression. In China, DKD treatment relies on administration of integrated Chinese and Western medicines as a common treatment strategy, with use of the injected traditional Chinese medicine astragalus in combination with ACEI/ARB achieving good clinical effect. However, most published systematic reviews that have evaluated astragalus injection as a DKD treatment have been limited to patients in a single disease stage, with no reviews focusing on randomized controlled trials. Therefore, no published systematic review exists that comprehensively describes clinical effectiveness and safety of combination therapy for the treatment of DKD, prompting our research group to conduct this study in spite of its unavoidable limitations. First, due to the fact that astragalus injection is a traditional Chinese medicinal preparation, it is mainly used in China and thus our focus on this drug may lead to regional results bias in selection of studies even in the absence of language-based restrictions. Consequently, such bias may lead to predominant selection of studies conducted in China, even though DKD is a globally prevalent disease. Nevertheless, while conducting this study our focus will be on alleviating patient suffering and thus we will not intentionally limit our research scope to studies conducted in countries where specific treatment drugs are manufactured. Ultimately, we hope this strategy will ensure that the results of this study will be applicable to diverse global populations in order to guide future development of a comprehensive treatment plan to benefit as many DKD patients as possible.

## Acknowledgments

The authors thank the National key research and development program of China for financial support.

## Author contributions

ZZ, WY and ZN conceived the study. ZZ and WY will develop the study protocol and implement the systematic review under the supervision of QZ and ZN. ZZ, LL and PB will design statistical analyses and carry out statistical analyses.SL and CS will perform literature search, screening, data extraction and risk of bias assessment. QZ and ZN will supervise the work. ZZ and WY wrote the first draft of the manuscript. All authors contributed to the drafting of the final protocol.

**Conceptualization:** Zhiyue Zhu, Wenbo Yang.

**Formal analysis:** Le Liu, Pengjie Bao.

**Funding acquisition:** Zheng Nan.

**Resources:** Shilin Liu, Chaoqun Song.

**Supervision:** Qi Zhang, Zheng Nan.

**Writing – original draft:** Zhiyue Zhu.

**Writing – review & editing:** Wenbo Yang.
